# York County Hospital

**Published:** 1913-06-14

**Authors:** J. Gordon Macqueen


### THE EDITOR'S LETTER-BOX.

### York County Hospital.

*To the Editor of* The Hospital.

Sir,—The report of Sir Cooper Perry and Mr. Basil Watson on the inquiry at the York County Hospital has now been issued, and appeared in The Hospital of last week. It clearly shows, as did the inquiry itself, the unbiassed nature and impartiality of those who conducted that inquiry.

Reviewing the whole circumstances, it may be said that in such an inquiry there were difficulties on both sides. For the York Hospital it was said that they did not know the specific nature of some of the charges, and therefore had not time to make a defence. As against that, it may be said that my colleague and myself had only two days' notice of the inquiry taking place, and whereas the York Hospital authorities had been going over the evidence for weeks beforehand (as the sheaves of typewritten manuscript produced at the inquiry showed), I only saw my counsel for the first time one hour and a half before the inquiry started. Again, it was endeavoured by the hospital authorities to show that in the case of "alleged ill-treatment of a child" the evidence for the charge had a general bearing on the whole attitude of my colleague and myself throughout all the charges; and as the tables were turned upon them (the hospital authorities), so to speak, it might be said that their evidence on that case had a general bearing on their attitude in answering all the charges.

Some of the charges are said to be unsubstantiated, and in regard to this I may say that there was evidence in the possession of Dr. Shepherd and myself relating to some of those charges which we had not permission to use, and it was impossible to obtain that permission in the two days' notice given to us.

Referring to the report itself :

Par. VI.—"Patient removed to the mortuary."

Here the charge was fully proved—viz., that the patient was removed to the mortuary alive, and that the "death" was not reported until after the removal of the body. It is clearly stated in the report that Dr. Shepherd was *not* guilty of any dereliction of duty in not seeing the patient when told by the night nurse of his condition. Had he seen him then, he would have given the same orders as he did without seeing him, and there is no proof that his orders would even then have been carried out or that the assumed death would have been reported.

Bearing the facts of the case clearly in mind, I have no hesitation in saying we were not interrupted at lunch, and that the removal took place before we had lunch. The question of time there also arose, and only served to show how long it took to prepare a bed in the emergency (isolation) ward.

Par. VIII.—"The treatment of surgical shock."

The report says: "It is most unlikely that such an operation should have been permitted to a house surgeon alone." To my personal knowledge it was not the only occasion that the same house surgeon performed a laparotomy followed by resection of gut.

Even though the time which the patient was left unattended was limited to "not more than five minutes," and even although no harm did come to the patient, five minutes is of sufficient length to enable a patient "coming out of an anæsthetic" to fall off a narrow trolley with disastrous consequences after such an operation.

Par. X.—"The Slipper Case."

In the report there appears "the sister affirmed that she told the nurse to get the slippers." It is unfortunate that the nurse's story and that of the patient concerning this were not obtained—a story which I believe is to the effect that the sister afterwards said that the patient would do as she was.

Par. XI.—"Alleged inefficiency of a 'special' nurse."

In regard to this it may be said that the complaint was not that the nurse was inefficient, but was rather a complaint against the matron that, considering the severity of the case, she did not choose the most experienced nurse which she had.

Par. XXIV.—"The treatment of sick nurses."

"(*a*) A case of diphtheria."—It was shown in evidence that the nurse reported herself as feeling unwell on October 1, took to bed on October 2, and on October 3 was reported to and seen by Dr. E., and on that evening had antitoxin administered—evidently, to my mind, not until *the third day* of the disease, and as it was not until this day also that she was reported to Dr. E., surely it is clear proof of the complaint that there was a failure on the part of the heads of the nursing staff to report *immediately* to the medical staff illnesses of nurses.

Case (*c*) brings out the fact that sick nurses were left in their bedrooms in the month of December without a fire. It seems to me that such treatment, no matter how trivial the ailment, would not lead to their comfort or be the means of producing a speedy recovery.

(*d*) "A case of abdominal colic."—As regards this case, it was unfortunate that the nurse's evidence could not be obtained; but I think that the alleged triviality of the complaint is negatived by the fact that the nurse has not yet returned to duty—over four months since her departure from the hospital.

As regards the other charges—most of them proved—I need say nothing here, save that if, by being the means of this inquiry, any good will accrue to the suffering poor and the sick nurses of York, my colleague and myself will feel amply repaid for the work which we have done.

In conclusion, I wish to express my thanks to The Hospital and to its Editor for the great part which they have taken in furthering this cause.—I am, Sir, yours faithfully,
J. Gordon Macqueen.

June 9, 1913.

